# Unique Scabies Presentation in a Patient with Bullous Pemphigoid

**DOI:** 10.4269/ajtmh.24-0113

**Published:** 2024-07-16

**Authors:** Delin Ran, Fangfang Bao, Donghong Du

**Affiliations:** Hospital for Skin Diseases, Shandong First Medical University; Shandong Provincial Institute of Dermatology and Venereology, Shandong Academy of Medical Sciences, Jinan, Shandong, China

A 73-year-old man sought evaluation for a pruritic, erythematous perineum lesion and hyperkeratotic crusted plaques involving the glans penis for 15 days. The physical examination was significant for perineal erythema with red papules, scrotum flushing, and hyperkeratotic crusted plaques involving the glans penis (Figure 1A). Because he was diagnosed with bullous pemphigoid (BP) 5.5 month earlier by histopathology (Figure 1C) and immunofluorescence microscopy (Figure 1D), the likely diagnosis was a BP resurgence. However, BP was stable with oral methylprednisolone tablets (24 mg/day), the primary BP lesions had resolved, and the autoimmune serologic profile and serial BP antibody testing (BP180 and BP230) were persistently negative. It is worth noting that combined therapy of methylprednisolone with mycophenolate mofetil was used for 4 months during BP treatment, and mycophenolate mofetil was discontinued 1 month before the current onset. A microscopic examination of the scrapings from lesions in glans penis demonstrated mites (*Sarcoptes scabiei*; Figure 1B). Ultimately, a diagnosis of scabies was made. Treatment with 10% sulfur ointment was effective after 3 days.

**Figure 1.**
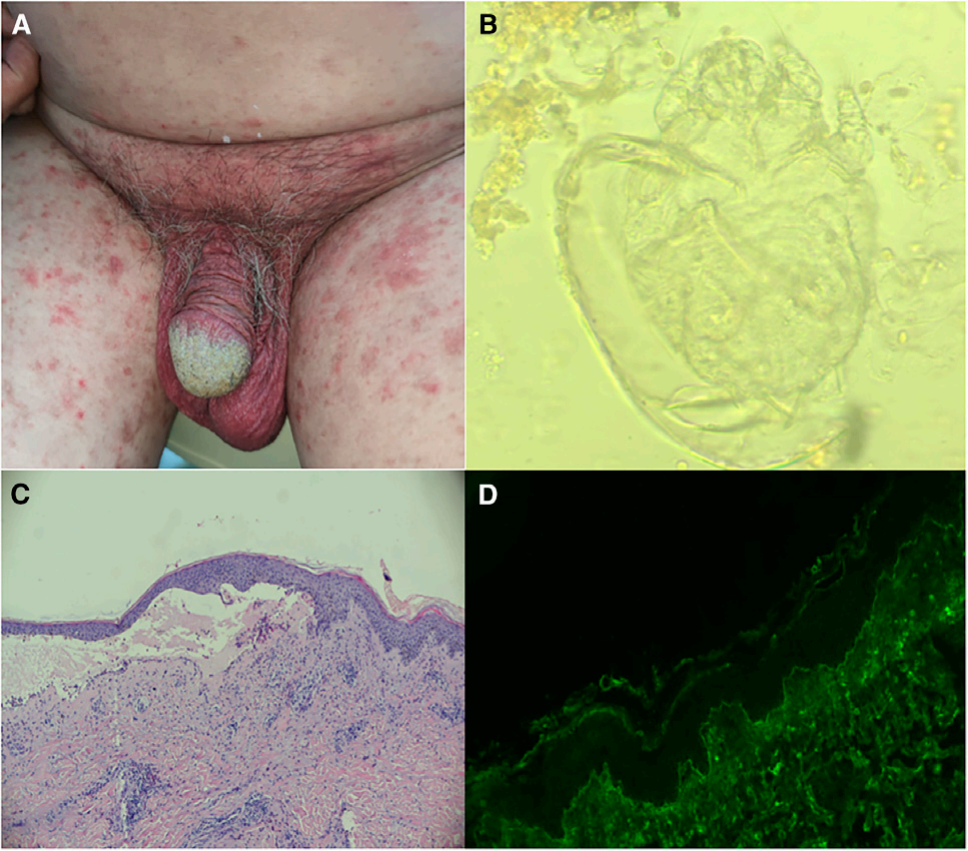
(**A**) Perineal erythema with red papules, scrotum flushing, and hyperkeratotic crusted plaques involving the glans penis. (**B**) Histopathology showed a subepidermal vesicles with abundant eosinophil infiltration in the papillary dermis. (**C**) Direct immunofluorescence showed a clear band of IgG and C3 deposition at the dermoepidermal junction (hematoxylin and eosin [HE] 40×). (**D**) microscopic examination of the scrapings from lesions in glans penis demonstrated mites (HE 100×).

Scabies is a skin infestation caused by a host-specific human scabies mite.^1^ BP is an autoimmune subepithelial bullous disease. However, the occurrence of scabies in patients with BP is relatively rare. A retrospective study revealed that 15 of 87 (17.2%) BP patients were diagnosed with scabies within 3 years of the initial BP diagnosis compared with 4.2% (11 of 261) of patients in the control group, which suggested an association between BP and scabies, but the basis for the association has not been established.^2^ In our patient with BP, we think prolonged use of methylprednisolone and mycophenolate mofetil may reduce the body’s immune system and increase susceptibility to infestation of *S. scabiei*. It is interesting to note that scabies lesion characteristics in glans penis were unusual.

## Data Availability

The data that support the findings of this study are available from the corresponding author, D. Du, upon reasonable request.
